# Insights into *Klebsiella pneumoniae* type VI secretion system transcriptional regulation

**DOI:** 10.1186/s12864-019-5885-9

**Published:** 2019-06-18

**Authors:** Victor Augusto Araújo Barbosa, Leticia Miranda Santos Lery

**Affiliations:** Cellular Microbiology Laboratory, Oswaldo Cruz Foundation - Oswaldo Cruz Institute, Av. Brasil, 4365 - Manguinhos, Rio de Janeiro, RJ CEP: 21040-900 Brazil

**Keywords:** Type VI secretion system, T6SS, *Klebsiella pneumoniae*, Transcriptional regulator; bacterial genome analysis

## Abstract

**Background:**

*Klebsiella pneumoniae* (KP) is an opportunistic pathogen that mainly causes respiratory and urinary tract infections. The frequent occurrence of simultaneously virulent and multiple drug-resistant isolates led WHO to include this species in the list of top priorities for research and development of therapeutic alternatives. The comprehensive knowledge of the molecular mechanisms underlying KP virulence may lead to the proposal of more efficient and specific drugs. One of its virulence factors is the Type VI Secretion System (T6SS), which contributes to bacterial competition, cell invasion and in vivo colonisation. Despite the few studies showing the involvement of T6SS in KP pathogenesis, little is known concerning the regulation of its expression. The understanding of regulatory mechanisms may give more clues about the function of the system and the possibilities of future interference in this process. This work aimed to standardise the annotation of T6SS genes in KP strains and identify mechanisms of their transcriptional regulation through computational predictions.

**Results:**

We analyzed the genomes of Kp52.145, HS11286 and NTUH-K2044 strains to perform a broad prediction and re-annotation of T6SS genes through similarity searches, comparative and linear discriminant analysis. 38 genes were found in Kp52.145, while 29 in HS11286 and 30 in NTUH-K2044. Genes coding for iron uptake systems are encoded in adjacencies of T6SS, suggesting that KP T6SS might also play a role in ion import.

Some of the T6SS genes are comprised in syntenic regions. 17 sigma 70-dependent promoter regions were identified in Kp52.145, 12 in HS11286 and 12 in NTUH-K2044. Using VirtualFootprint algorithm, binding sites for 13 transcriptional regulators were found in Kp52.145 and 9 in HS11286 and 17 in NTUH-K2044. Six of them are common to the 3 strains: OxyR, H-NS, RcsAB, GcvA, Fis, and OmpR.

**Conclusions:**

The data presented herein are derived from computational analysis. Although future experimental studies are required to confirm those predictions, they suggest that KP T6SS might be regulated in response to environmental signals that are indeed sensed by the bacteria inside the human host: temperature (H-NS), nutrition-limitation (GcvA and Fis), oxidative stress (OxyR) and osmolarity (RscAB and OmpR).

**Electronic supplementary material:**

The online version of this article (10.1186/s12864-019-5885-9) contains supplementary material, which is available to authorized users.

## Background

The Type Six Secretion System (T6SS) was initially described when it was demonstrated that secretion of the Hcp (Hemolysin-Coregulated Protein) and VgrG (Valine-Glycine Repeats) proteins were independent of the other known secretion mechanisms [1, 2]. In pathogenic bacteria, secretion systems can be used at various stages of the bacterial infection pathway, such as toxin export, cell adhesion and direct translocation of effectors into the host cell or to delivery toxins against competitor bacteria [3]. A great diversity of these effectors and toxins have been identified and associated with the T6SS, which makes it a versatile weapon [4–6].

A functional T6SS apparatus is composed of the products of at least 13 conserved genes (*tssA-M*) [7] . The T6SS components assemble in a contractile needle-shaped apparatus which translocates effectors to neighboring cells [8]. It comprises a transmembrane complex, attached to a baseplate complex and a long-tailed cytoplasmic tubular structure surrounded by a contractile sheath. The contraction of the sheath propels the inner tube through the membranes towards the target cell. Effector proteins are carried by the T6SS tip and/or tubular components and are released after the tube disassembly inside the target cell.

In silico analyses have demonstrated the presence of T6SS genes in several Gram-negative bacteria, with the numbers of orthologs varying in each bacterium [7, 9–11]. Besides, the genomes of some bacteria can encode the set of T6SS genes in more than one *locus* [7, 12]. Commonly, these *loci* are within pathogenicity islands - for instance, the *Pseudomonas aeruginosa* HSI (Hcp-secretion island) and the *Salmonella typhimurium* SCI (*Salmonella* centrisome island), as well as in the genomes of enteroaggregative *Escherichia coli*, and *Vibrio cholerae* [4, 12–14] . In addition to the genes encoding the 13 conserved components of T6SS, these genomic *loci* may encode toxins, antitoxins, adapters, and auxiliary proteins, as well as additional effectors [4, 7] .

In another hand, some functional T6SS genes are found outside the referred genomic islands: they are referred to as T6SS “orphan” genes [12]. Therefore, identifying these genes in bacterial genomes is also crucial for understanding the functionality of T6SS.

Besides, bacteria may encode several functional ‘copies’ of each T6SS gene. The expression of the different isoforms of a component may vary according to the target cell and/or by the environmental condition in which the bacterium is sensing [5, 13]. The expression of these secretion complexes can be precisely regulated by transcriptional, translational and post-translational mechanisms [15, 16] .

*Klebsiella pneumoniae* (KP) is a ubiquitous species in nature, a gut commensal, and a human opportunistic pathogen. It can cause a wide range of infections, including pneumonia, urinary tract infections, bacteremia and liver abscesses [17, 18]. Due to the frequent occurrence of multiple antibiotic-resistant isolates, *K. pneumoniae* is considered a global public health concern [19–21] . Capsule, fimbriae, lipopolysaccharide (LPS) and siderophores are important and well-characterized virulence factors from KP [17]. Recently, additional factors have been described, such as type II (T2SS) and type VI secretion systems (T6SS) [22–25]. However, there is significant heterogeneity among KP strains and those virulence factors may play different roles in different strains [17, 26].

So far, little is known about T6SS in *K. pneumoniae*. From a genomic perspective, genes coding for putative T6SS components are present in KP genomes. In some strains, T6SS genes are grouped mainly in 2 *loci* (for instance: NTUH-K2044, Kpn2146 and HS11286), while in others they are found in 3 *loci* (Kp52.145, MGH 78578, 342) [22, 24, 27]. As KP genomes were annotated using different approaches - some using automatic pipelines - there is no uniform annotation of T6SS genes. In a functional perspective, Lawlor and colleagues (2005) screened a transposon library and found that 2 mutants in hypothetical protein-coding genes displayed decreased ability to infect mouse spleen [28]. Those proteins were later annotated as putative T6SS components. Recently was described that HS11286 strain T6SS secretes a phospholipase effector with antibacterial activity [24]. Moreover, it has been shown that T6SS mutants in NTUH-K2044 strain significantly reduced bacterial killing, the expression of type-1 fimbriae and adherence and invasion of epithelial cells [25]. Concerning T6SS regulation, it has been observed that strain Kp52.145 genes are expressed when the bacterium colonized mice lungs, but not when the bacterium grows in TCS culture medium [22]. In Kpn2146, an RNA-seq analysis revealed that most of the T6SS genes increased expression 24 h post-infection of macrophages [29]. In HS11286 was suggested that sub-inhibitory concentrations of antibiotics might regulate T6SS secretion [24]. In NTUH-K2044 strain, was demonstrated that histone-like nucleoid structuring protein (H-NS) binds regulatory region and inhibits *tssD* (Hcp/tube component) expression [25].

Based on growing evidence for T6SS relevance for KP pathogenesis and divergent genomic features of T6SS loci between KP strains, this work aimed to identify every T6SS gene in 3 KP strains and standardize T6SS gene annotation in KP. Moreover, we hypothesized that translational mechanisms could be involved in T6SS expression in KP. Therefore, we predicted transcriptional regulator’s binding sites upstream transcriptional start sites for T6SS genes and obtained insights into T6SS role and regulation.

## Results

### Identification of T6SS genes in KP genomes

Previous studies have identified T6SS genes in KP genomes. Sarris and colleagues (2011) annotated T6SS genes in three fully sequenced KP strains (342, NTUH-K2044 and MGH78578) and one partially sequenced strain (KP subsp. rhinoscleromatis ATCC13884) [27]. However, at that time there were few KP complete genomes available for comparison. In this study we present a similar effort. However, we focused on the human pathogenic strains: Kp52.145 (a K2 virulent strain), HS11286 (a multiple-drug-resistant strain) and NTUH-K2044 (a K1 virulent strain). Besides, we propose herein the use of the TssA-M nomenclature for the T6SS core components in KP. This nomenclature overcome the problem that T6SSs in different organisms had historically acquired different, system-specific names for equivalent components [7]. Thus, the genomes of the 3 KP strains mentioned above were reanalyzed to identify and re-annotate all putative T6SS protein-coding genes (*tssA-M*, *paar, tagA* and *tagL*). The starting point for such annotation process was the analysis provided by SecreT6 database. Then, VRProfile, Blast, COG, PFAM and CDD searches were also performed (Additional file 1). The same search and filtering criteria were applied to the 3 genomes.

In overall, we detected 38, 29 and 30 genes possibly related to T6SS in the Kp52.145, HS11286, and NTUH-K2044 genomes, respectively (Table 1).

**Table 1:** T6SS components in *K. pneumoniae* strains Kp52.145, HS11286 and NTUH-K2044. Locus tag for each gene is shown

	Kp52.145	HS11286	NTUH-K2044
*tssA*	BN49_RS05995; BN49_RS14080; BN49_RS18715	KPHS_23150; KPHS_32450;	KP1_RS11300; KP1_RS15690;
*tssB*	BN49_RS14020;	KPHS_22970;	KP1_RS11220; KP1_RS11085;
*tssC*	BN49_RS14025;	KPHS_22980;	KP1_RS11225;
*tssD*	BN49_RS06500; BN49_RS15545;	KPHS_23020; KPHS_41670;	KP1_RS11245; KP1_RS12525;
*tssE*	BN49_RS05940; BN49_RS18720;	KPHS_32460;	KP1_RS15695;
*tssF*	BN49_RS05970; BN49_RS14085; BN49_RS18735;	KPHS_23170; KPHS_32490;	KP1_RS11315; KP1_RS15710;
*tssG*	BN49_RS05965; BN49_RS14090; BN49_RS18730;	KPHS_23180; KPHS_32480;	KP1_RS11320; KP1_RS15705;
*tssH*	BN49_RS14050; BN49_RS07300; BN49_RS08275; BN49_RS11635;	KPHS_23030; KPHS_39850; KPHS_17930; KPHS_11410;	KP1_RS11250; KP1_RS19445; KP1_RS06015; KP1_RS08880;
*tssI*	BN49_RS06025; BN49_RS18800;	KPHS_23040; KPHS_32730;	KP1_RS11255; KP1_RS15775;
*tssJ*	BN49_RS05945; BN49_RS14095; BN49_RS18725;	KPHS_32470;	KP1_RS11325; KP1_RS15700;
*tssK*	BN49_RS06040; BN49_RS14030; BN49_RS18815;	KPHS_22990; KPHS_32770;	KP1_RS11230; KP1_RS15790;
*tssL*	BN49_RS06035; BN49_RS14035; BN49_RS18810;	KPHS_23000; KPHS_32760;	KP1_RS11235; KP1_RS15785;
*tssM*	BN49_RS06000; BN49_RS14075;	KPHS_23140; KPHS_32500;	KP1_RS11295; KP1_RS15715;
*Paar*	BN49_RS05990; BN49_RS14070; BN49_RS18750;	KPHS_23120; KPHS_32520;	KP1_RS15725; KP1_RS11305;
*tagL*	BN49_RS06030; BN49_RS14040; BN49_RS18805;	KPHS_23010; KPHS_32750;	KP1_RS11240; KP1_RS15780;

#### Kp52.145

Its genome annotation has been updated recently (FO834906.1 from 07-MAR-2015/NZ_FO834906.1 from 21-FEB-2017). We identified that six T6SS-related genes identified by SecreT6 in the initial genome version were re-annotated as pseudogenes in the most recent version (BN49_RS18740, BN49_RS14045, BN49_RS14055, and BN49_RS05960) - therefore, they do not seem to code for T6SS components and indicate that these genes may be under genome reduction process.

In agreement with previous study by Lery et al. (2014), we found that most of the T6SS genes in Kp52.145 genome (33 genes, 87%) are clustered in 3 genomic loci and only 5 (12%) are orphan genes: *tssH* (BN49_RS07300, BN49_RS08275, BN49_RS11635) and *tssD* (BN49_RS06500 e BN49_RS15545). Apart from the genes identified in this previous study, we found 7 additional putative T6SS: 1 gene coding for a PAAR domain-containing protein, 1 gene coding for an OmpA-family protein, 3 genes encoding putative TssH (Clp ATPases) components and 2 genes coding for TssD components. All the 38 putative T6SS-related genes in Kp52.145 genome are described in Additional file 2.

#### HS11286

A previous study revealed a 23-gene T6SS cluster (KPHS_22970 to 23,190) on the chromosome of KP HS11286, containing 12 core T6SS components [24]. In our analysis we found 25 genes annotated as T6SS-related by SecreT6. Four additional genes were identified by further analysis: 3 genes encoding putative TssH components (KPHS_39850, KPHS_17930, KPHS_11410) and one gene encodes a TssD component (KPHS_41670). 25 out of the 29 (86%) T6SS-genes are clustered in two main *loci* and only 4 (14%) are orphan genes: 3 *tssH* and 1 *tssD*. All the 29 putative T6SS-related genes in HS11286 genome are described in Additional file 2.

#### NTUH-K2044

25 genes were annotated as T6SS-related by SecreT6. Besides those genes, we found 3 genes encoding putative TssH components, 1 TssB and 1 TssD that might be part of T6SS in this strain. Accordingly to a previous study, most of the genes (25 genes, 83%) are clustered in 2 loci, and only 5 genes (17%) are orphan: *tssB* (KP1_RS11085), *tssD* (KP1_RS12525), *tssH* (KP1_RS19445, KP1_RS06015, KP1_RS08880). All the 30 putative T6SS-related genes in NTUH-K2044 genome are described in Additional file 2.

### Annotation of Clp ATPases: putative TssH components

ClpV ATPases have been initially described as the TssH member of T6SS complex, required for a functional tube formation and recycling of sheath components [30–32]. ClpV proteins in KP genomes are encoded by genes BN49_RS14050 in Kp52.145, KPHS_23030 in HS11286 and KP1_RS11250 in NTUH-K2044. Kp ClpV proteins are 99% identical.

Recently has been shown that *Francisella tularensis* has a non-canonical functional T6SS that uses ClpB ATPase instead of ClpV [33, 34]. Interestingly, the *clpB* gene KP1_RS19445 (former KP1_4170) from KP NTUH-K2044 has been associated to T6SS [27]. Therefore, we expanded the search for Clp ATPases in KP genomes - as they could be putative TssH. In addition to *clpV* genes, SecreT6 and VRprofile predicted 3 additional Clp-family ATPases, belonging to COG0542, encoded in each KP genome analyzed: ClpB, ClpA e ClpX.

**Fig. 1:**
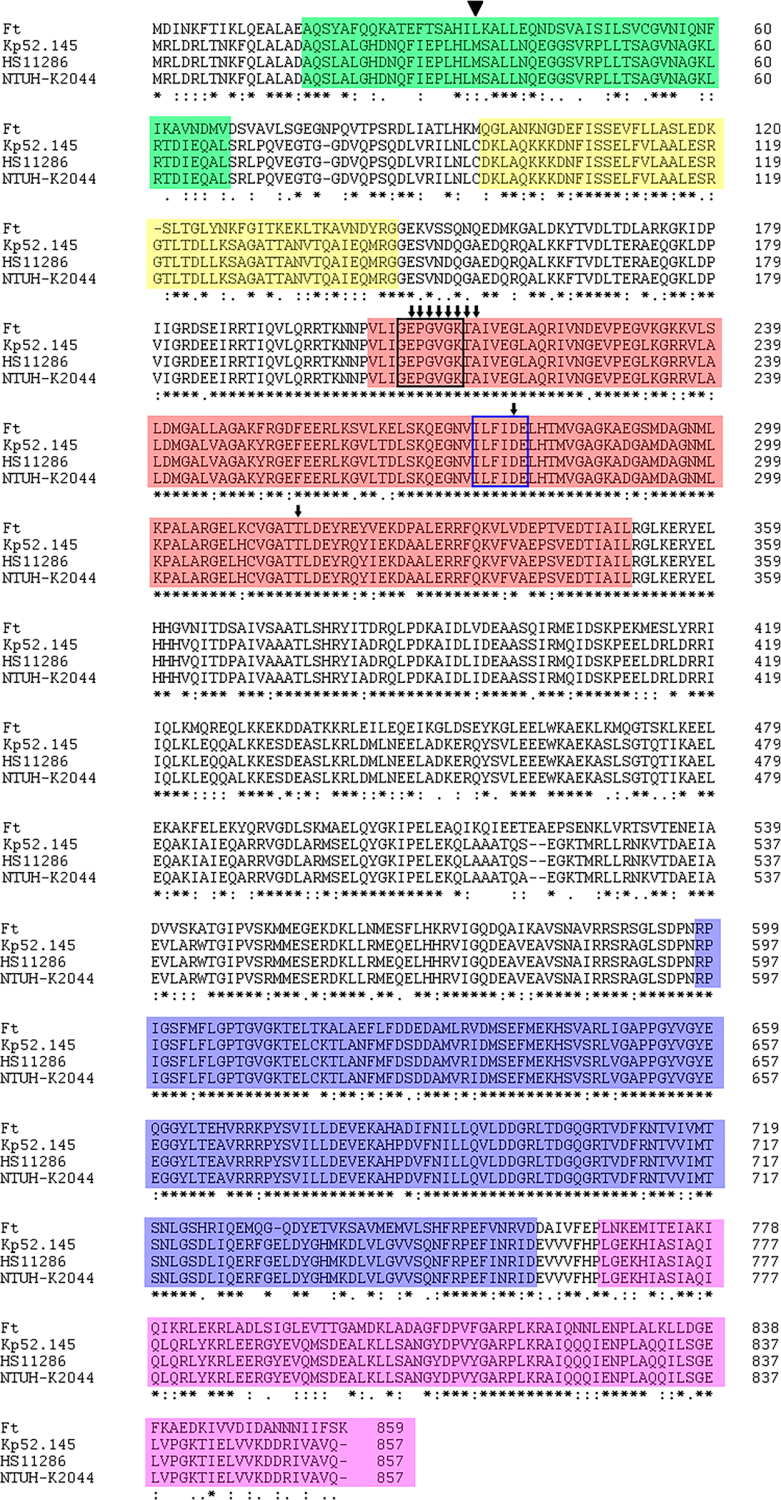
KP ClpB proteins are quite similar to *F. tularensis* ClpB: Multiple sequence alignment of ClpB proteins from *F. tularensis* SCHU (Ft), *K. pneumoniae* Kp52.145, HS11286 and NTUH-K2044. Green and yellow regions indicate the Clp_N domain; Red, blue and purple are the AAA, AAA2 and ClpB_D2-small domains, respectively. Black and blue boxes indicate the Walker **a** and Walker **b** domains; Arrows point to ATP binding site residues. The arrow head indicate the position 1 of ClpB from HS11286 according to its previous annotation. “*”Identity of all aminoacids in the indicated column, “:”Similarity of aminoacids aligned, “.” Low similarity of aminoacids aligned, according to ClustalW default parameters

According to the deposited genome annotation, Kp52.145, HS11286 and NTUH-K2044 ClpB proteins would be 857, 823 and 857 aminoacids long. According to our analysis, the start codon for HS11286 *clpB* has been previously misannotated. We suggest it is 102 bp longer, coding a protein of 857 aminoacids. Considering such re-annotation, protein sequences from the 3 KP strains are 100% identical. KP ClpB protein is quite conserved to *F. tularensis* ClpB: 64% sequence identity and 79% similarity (Fig. 1). In addition, KP ClpB presents the same conserved domains as its *F. tularensis* orthologue: Clp_N, ClpB_D2small, AAA and AAA2 domains, as well as Walker A, Walker B, ATP binding site and arginine finger motifs - suggesting that they might be able to perform similar roles. Further studies are required to confirm it.

ClpA proteins from the 3 KP strains share 99% identity. In comparison to ClpV sequence they are ~ 53% similar and 35% identical in an alignment of 87% coverage. Despite such sequence dissimilarities, ClpA, ClpB and ClpV contain conserved domains (Additional file 3). The KP ClpX sequences are identical among the 3 strains, however ClpX is 424 aminoacids long, while ClpV is 884. Whether ClpA and ClpX could play the role of TssH member at T6SS complex remains to be elucidated.

### Genomic context of T6SS genes

Bacterial genes required for the same functional process are often clustered in the same genomic region and frequently subjected to the same regulatory network. As mentioned above, most of the KP T6SS genes are clustered in 2 or 3 regions per genome, presenting characteristics of genomic islands (GC content different from the average genome, inserted in tRNA loci, containing transposase or other mobile elements). None of those islands encode any transcriptional regulator. We analysed the function of the gene products neighbouring T6SS genes to get insights into processes that could be co-regulated and identify putative regulatory proteins. Curiously, we found genes encoding conserved LysR-type transcriptional regulators from superfamily PBP2 ~ 3 to 4 kb from several T6SS *loci* (Table 2).

**Table 2:** Transcriptional regulators predicted to be involved in KP T6SS regulation

Transcriptional regulator	Locus-Tag			T6SS relationship*
	Kp52.145	HS11286	NTUH-K2044	
Fis	BN49_RS03455	KPHS_48020	KP1_RS23255	Binding sites predicted at 17, 12, 12 promoters
OxyR	BN49_RS25290	KPHS_01030	KP1_RS00535	Binding sites predicted at 15, 10, 12 promoters
H-NS	BN49_RS18480	KPHS_31980	KP1_RS15455	Binding sites predicted at 13, 8, 10 promoters
OmpR	BN49_RS02915	KPHS_27610	KP1_RS23790	Binding sites predicted at 9, 6, 7 promoters
GcvA	BN49_RS13685	KPHS_42400	KP1_RS10795	Binding sites predicted at 8, 3, 6 promoters
RcsAB	BN49_RS21260	KPHS_37040	KP1_RS18010	Binding sites predicted at 3, 4, 5 promoters
LysR_PBP2	BN49_RS18845	KPHS_32830	KP1_RS15820	Encoded 3058 bp, 3059 bp, 3059 bp from T6SS cluster
	BN49_RS13995	–	KP1_RS11195	Encoded 4025 bp, −, 4024 bp from T6SS cluster

* the numbers indicated correspond to strains Kp52.145, HS11286 and NTUH-K2044, respectively

**Fig. 2:**
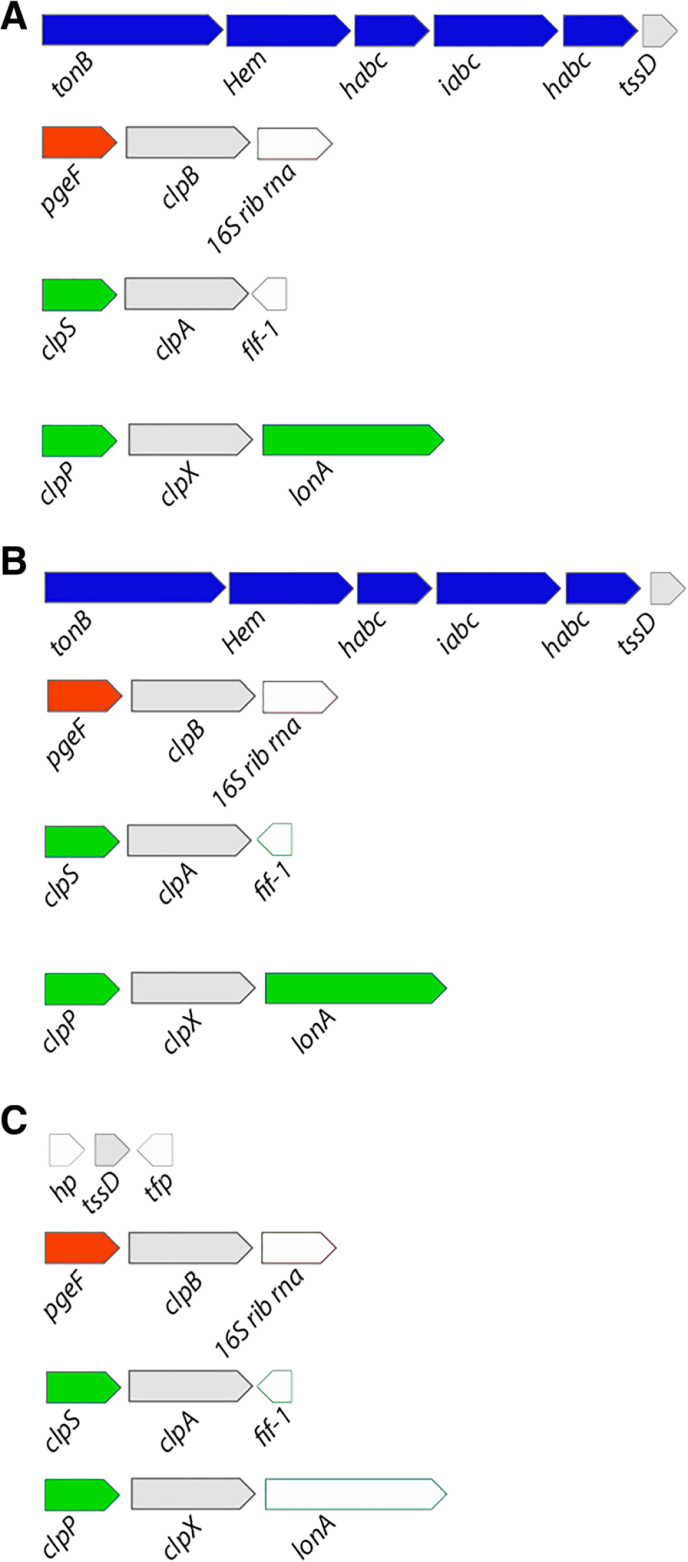
Genes coding for iron-related transporters are adjacent to *tssD* in KP genomes. Genomic context analysis of T6SS orphan genes in Kp52.145 (**a**), HS11286 (**b**) and NTUH-K2044 (**c**). T6SS-related genes are colored in gray; genes coding for iron uptake-related proteins are in blue; proteases, peptidases and ATPases are in red; genes encoding other functions are represented in white

The 3 genomes also presented non-clustered T6SS-related ´orphan´ genes. We found outer membrane ion transporters encoded in genes neighboring orphan *tssD* genes (Fig. 2). In Kp52.145, the *tssD* encoded by BN49_RS06500 is located in a region encoding hypothetical proteins, iron/heme ABC-family transporters (BN49_RS06505 to BN49_RS06520) and a TonB-dependent receptor (BN49_RS06525). In HS11286 the *tssD* orphan gene (KPHS_41670) neighbors genes that encode an hemin ATP transport system (KPHS_41660 to KPHS_41640), an S-adenosylmethionine-dependent methyltransferase (KPHS_41630) and an outer membrane receptor for ferric enterobactin and colicins B and D (KPHS_41620). Although these iron-related genes in Kp52.145 and HS11286 have a different names, they are orthologs and present the same sequence. Thus, both orphan *tssD* are encoded in the same genomic region. Interestingly, recently has been shown that in response to oxidative stress, T6SS from *Yersinia pseudotuberculosis* and *Burkholderia thailandensis* secrete effectors involved in ion uptake. The ion import is further mediated by ABC and/or TonB-family proteins [35–37].

In the neighborhood of the putative *tssH*/*clpB* (BN49_RS07300 in Kp52.145 and KP1_RS19445 in NTUH-K2044) there is *pgeF -* coding for a peptidoglycan editing factor. PgeF contributes to the maintenance of the peptidoglycan peptide chain composition in *E. coli*, thus contributing to the integrity of the bacterial peptidoglycan layer [38]. Moreover, in the neighborhood of *clpX*, another putative *tssH* (BN49_RS08275, KPHS_11410 and KP1_RS06015) we found a *clpP* ATPase and the LonA endopeptidase; and besides the *clpA tssH* (BN49_RS11635, KPHS_17930 and KP1_RS0880), a *clpS* ATPase and macrolide transport proteins. Further experiments will be performed to check whether these gene products could be related to or secreted by T6SS, used for bacterial competition and/or tissue invasion.

### Synteny among T6SS loci in the 3 strains

**Fig. 3:**
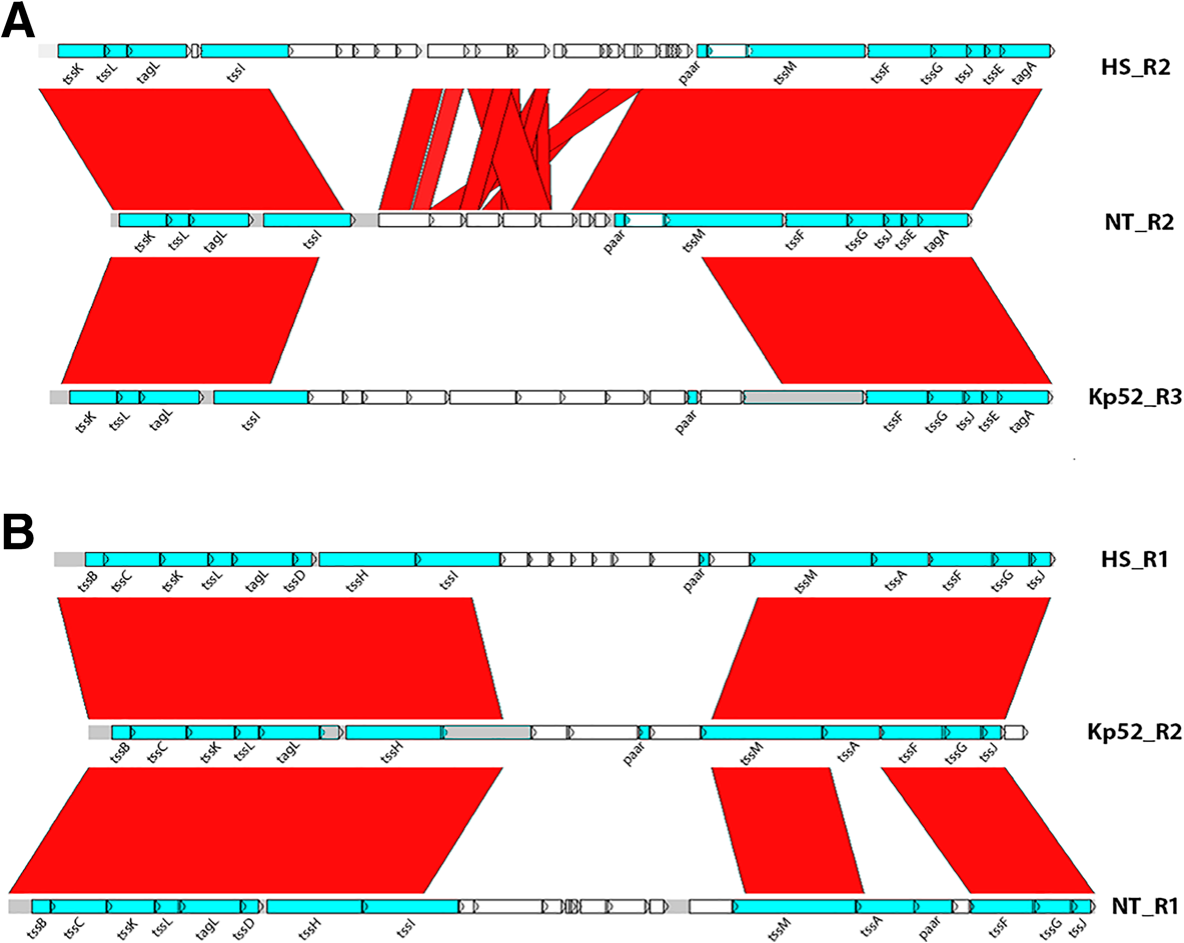
Comparative analysis of genomic regions encoding T6SS-related genes in *K. pneumoniae* strains Kp52.145 (Kp52), NTUH-K2044 (NT) and HS11286 (HS). Genomic regions in each genome were named R1, R2 and R3, according to their genomic locations. BLASTN analysis was performed using DoubleACT 2.0 and displayed with the ACT software. T6SS-related genes are represented in blue arrows, non-T6SS genes in white. Pseudogenes are displayed in gray. Regions of synteny between the sequences are displayed in red blocks

We analyzed the synteny and sequence conservation among the genomic regions coding for T6SS genes in Kp52.145 (Kp52_R1; Kp52_R2; Kp52_R3), HS11286 (HS_R1; HS_R2) and NTUH-K2044 (NT_R1; NT_R2). Pairwise alignments of the entire regions coding for T6SS genes were visualized in ACT software. Regions of similarity and differences among the strains are pointed in Fig. 3. Two distinct regions > 24 kB were aligned among the 3 genomes (Fig. 3, a and b). In both regions, genes annotated as T6SS-related are encoded in the beginning and end of those sequences, while the middle regions contain genes coding for other functions. Curiously, the T6SS-related genes are syntenic among the 3 strains. Syntenic regions with > 99% sequence identity code for *tssK-tssL-tagL-tssI and paar-tssM-tssF-tssG-tssJ-tssE*-*tssA* (Fig. 3a) */ tssB-tssC-tssK-tssL-tagL-tssD-tssH-tssI and tssM-tssA-tssF-tssG-tssJ* (Fig. 3b). It is worthy to note that *tssD* and *tssI* at Kp52_R2 and *tssM* in KP52_R3 are currently annotated as pseudogenes.

In another hand, the insertions containing T6SS-unrelated genes are less conserved or strain-specific, encoding mainly proteins of unknown function or transposases. Regions HS_R2 and NT_R2 share short regions of similarity varying from 87 to 96% identity. In another hand, the insertion observed in Kp52_R3 contains genes coding for phospholipases, Sel-1 lipoproteins and a PAAR protein. Such region has been previously characterized as implied in KP virulence [22].

### Transcriptional regulator’s binding sites

**Fig. 4:**
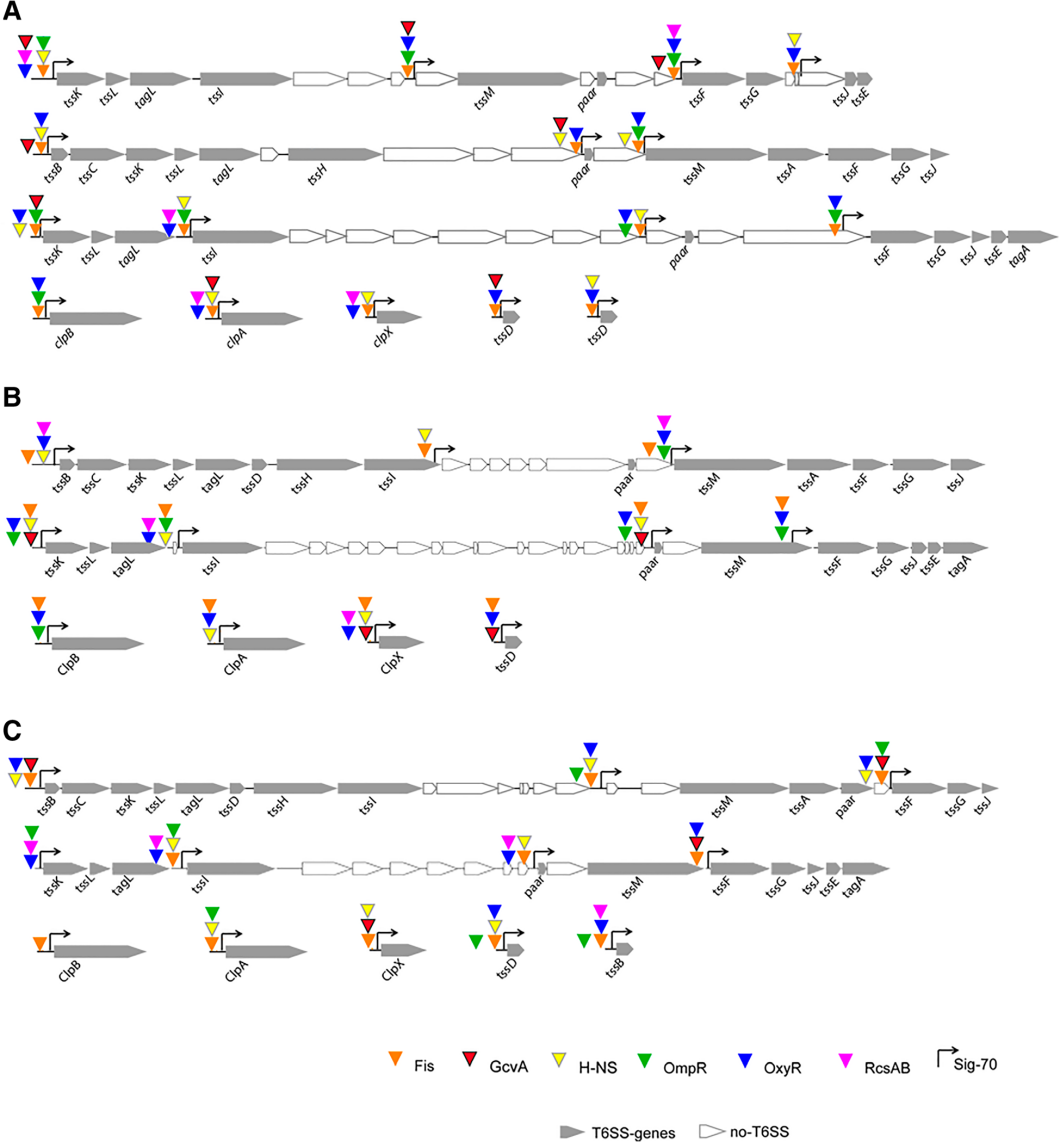
T6SS genes organisation, predicted transcriptional start sites and putative transcriptional regulators binding sites in *K. pneumoniae* Kp52.145 (**a**), HS11286 (**b**) and NTUH-K2044 (**c**) genomes. T6SS genes are represented as gray arrows. Colored triangles point the position for the 6 transcriptional regulators with more binding sites predicted

To get insights into T6SS transcriptional regulation in KP, we identified 휎70 promoter consensus sequences − 10 and − 35 upstream the CDSs coding for the T6SS-related genes (Additional file 4). In overall, 17 putative 휎70-dependent transcriptional start sites were found in Kp52.145 (Fig. 4a), 12 in HS11286 (Fig. 4b) and 12 in NTUH-K2044 (Fig. 4c). 250 bp upstream each of those transcriptional start sites were analyzed using position-weight matrices to identify putative binding sites (Additional file 5). In Kp52.145, 106 binding sites for 13 transcriptional regulators were predicted: Crp, CytR, FhlA, Fis, Fnr, GcvA, H-NS, MalT, MetR, Mlc, OmpR, OxyR and RcsAB (Additional file 6). In HS11286, there were 72 binding sites for 09 transcriptional regulators: Fis, GcvA, H-NS, Lrp, NarL, OmpR, OxyR, PdhR and RcsAB (Additional file 7). In NTUH-K2044 there were 114 binding sites for 17 transcriptional regulators: Crp, FadR, FhlA, Fis, Fnr, GcvA, GlpR, H-NS, Lrp, MalT, MetJ, MetR, Mlc, OmpR, OxyR, PdhR and RcsAB (Additional file 8).

Six of these regulators had binding sites upstream T6SS genes predicted in the three strains: Fis, OxyR, H-NS, OmpR, GcvA and RcsAB (Table 2) - indicating that to some extent there might be conservancy of regulatory mechanisms. Curiously, ~ 85% of the promoter sequences analyzed contain conserved Fis and OxyR binding sites, and ~ 75% for H-NS. All those putative binding sites described above were computationally predicted. Further studies are required to experimentally validate them.

## Discussion

KP virulence factors content and expression vary among different isolates. T6SS genes are largely distributed in KP strains. Previous studies identified T6SS orthologs in KP strains 342, NTUH-K2044, MGH78578, HS11286 and BAA2146 [24, 25, 27]. In this study we performed a robust computational analysis of T6SS genes and genomic context, as well as putative protein sequences to get more insights into T6SS role in KP. Comparing the genomes of 3 human pathogenic KP strains (NTUH-K2044, HS11286 an Kp52.145), the analysis presented herein allowed us to propose an standardization of T6SS genes nomenclature in KP. The results presented herein show that KP T6SS core genes encoded in the genome of 3 KP strains are somewhat conserved in terms of sequence similarity, gene content and operon structure. In addition, they are similar to the T6SS-encoding regions in *E. coli* E042 [4] and *Pantoea ananatis* [39]. Using the approach described above, we identified putative T6SS orthologs not described previously - specially putative TssH family proteins. TssH is a ClpV ATPase shown to be involved in T6SS disassembly and subunits recycling [30, 31] However, recently has been shown that other Clp/Hsp100 family proteins such as ClpB from *Francisella tularensis* might play a similar role [33, 34]. The approach performed in this study identified ClpB, ClpA, ClpX and ClpV proteins as putative TssH. Due to the high similarity between KP and *F. tularensis* ClpB, we propose that KP ClpB proteins might function as a TssH. However, whether those proteins are indeed required and/or assembled as part of T6SS in KP remains to be elucidated.

We observed that the majority of the T6SS genes in KP are clustered in 2 or 3 genomic islands - in agreement with Sarris et al. [27]. Interestingly, we identified genes coding for PAAR proteins in every T6SS island of the 3 strains. We observed that T6SS core genes are presented in syntenic blocks, whilst insertions of variable content are strain-specific. Those insertions mainly code for proteins of unknown function, but also for putative T6SS accessory or effector proteins. A functional relationship between T6SS and such genes inside insertions is provided by the transcriptomic analysis of Bent et al. showing the co-expression of some of those genes [29].

So far, KP T6SS had been associated to antibacterial activity, cell invasion and in vitro colonization [24, 25]. The genomic context analysis described herein pointed that several iron-related transporters are encoded around T6SS genes. This observation raises the hypothesis that KP T6SS could play a role in ion uptake. For instance, *Y. pseudotuberculosis* T6SS transports Zn^2+^ to Combat Multiple Stresses and Host Immunity [35]. *B. thailandensis* uses T6SS to uptake Mn^2+^ ions, to resist oxidative stress and compete with other bacteria [36] . Such *B. thailandensis* T6SS is regulated by OxyR, a conserved oxidative stress response transcriptional regulator. Reactive oxygen species (ROS) can damage bacterial cells, thus, bacteria detoxify ROS by producing ROS-detoxifying enzymes, DNA repair, and sequestration of metal ions. One of those mechanisms involves catalases. Curiously, OxyR regulates the expression of KatN*,* a catalase secreted in a T6SS-dependent manner by enterohemorrhagic *E. coli* [40]*.* OxyR is one of the regulators that induce the bacterial oxidative stress response. In addition, it has been demonstrated that KP OxyR regulates biofilm formation, fimbrial genes, antibiotic resistance and adhesion to epithelial cells [41, 42]. Interestingly, we predicted conserved OxyR binding sites in most of the KP T6SS promoter regions, in all 3 strains analyzed. Altogether, these data suggest that KP T6SS might be activated under oxidative stress conditions and might help the bacteria to deal with ROS detoxification.

Another interesting finding was that putative H-NS binding sites are widely present and conserved in KP T6SS promoters. It has been previously shown that H-NS silencing of a T6SS locus limits *Salmonella enterica* interbacterial killing [43]. H-NS is a nucleoid structuring protein with global effects on silencing gene expression [44]. Its activity depends on temperature and osmolarity. It has been shown that KP H-NS represses the expression of important virulence factors, such as type-3 pili and capsule [45, 46]. Recently, Hsieh et al. (2018) showed that H-NS binds to *tssD* promoter and silences *tssD* (KP1_RS11245) expression in NTUH-K2044. Thus, our prediction was confirmed by this recent experimental data.

Putative binding sites for Additional 4 regulators were predicted in promoter sequences of T6SS genes of all 3 strains: RcsAB, GcvA, Fis and OmpR. RcsAB is an unusual regulatory system that binds an *rcsAB box* and modulates KP *galF* gene, thus affecting capsule expression and virulence [47, 48]. GcvA is the transcriptional regulator of the glycine cleavage system, involved in aminoacids metabolism. GcvA has not yet been studied in KP, however, it is required for *F. tularensis* fitness and full virulence [49]. Fis (factor for inversion stimulation) is a transcriptional regulator that respond to changes in the nutritional environment in enterobacteria [50]. OmpR is the response regulator of a two-component system with the sensor kinase EnvZ. OmpR binds to the promoter region of a *Yersinia pseudotuberculosis* T6SS involved in the bacterial survival in high osmolarity conditions, resistance to deoxycholate and pH homeostasis [51, 52]. It has been shown that KP OmpR regulates c-di-GMP signaling pathway, type 3 fimbriae expression, and biofilm formation in response to osmotic stresses [53]. Altogether, these results comprise quite interesting data suggesting that KP T6SS may be regulated in response to environmental signals that are indeed sensed by the bacteria inside the human host: temperature (H-NS), nutrition-limitation (Fis), oxidative stress (OxyR) and osmolarity (RscAB and OmpR). Strikingly, H-NS, OxyR and OmpR regulators are expressed by KP BAA2146 during in vitro macrophages infection [29].

Besides, we found conserved genes encoding LysR transcriptional regulators (LTTR) containing PBP2-like substrate binding domains, in the adjacencies of almost every T6SS gene cluster in the three KP strains studied in this work. Their consensus binding sequences have not yet been determined, thus we did not manage to further predict if they may indeed regulate KP T6SS.

It is important to highlight that, so far, we analyzed only sigma 70-dependent promoter sequences. Probably, other sigma promoters are involved in T6SS transcriptional regulation. In fact, Hsieh et al. detected that, in NTUH-K2044, *tssB-tssC-tssK-tssL-tagL-tssD* form a single transcriptional unit and that *tssH* and *tssI* genes are independently transcribed [25]. In our analysis we detected the sigma 70-dependent promoter upstream *tssB*, but not *tssH* and *tssI*. Thus, *tssH* and *tssI* might have alternative promoters - reinforcing that it will be useful in the future to expand this analysis to other promoters.

Recently, Ho et al. (2014) proposed a model for T6SS assembly and activity [54]. In such model, the baseplate components (TssAEFGJKLM) that anchor the system through the bacterial membranes are the first to be assembled [47]. Then, the coating proteins (TssI and Paar), contributing to the overall stability of the apparatus, are recruited. In a third step, tube and sheath proteins (TssBCD) are assembled. At last, TssH ATPase is required. Interestingly, we have identified binding sites for RcsAB and OmpR – transcriptional regulators related to osmotic stress – in the promoters of all baseplate and coating coding genes, but in none of the other components. In another hand, we found that every gene coding for tube, sheath or ATPase component might be regulated by OxyR. Although those predictions are still to be confirmed and additional promoters and regulators are expected to be involved, we hypothesize that at least two signals are required for the expression of KP T6SS.

## Conclusions

We presented a genomic analysis of 3 KP strains and provided new insights into T6SS role and regulation: 1) T6SS components annotation was standardised among the strains, 2) considering that KP ClpB protein sequences are quite conserved to *F. tularensis* ClpB, we suggest that they may act as TssH, 3) it was hypothesized that T6SS in KP might play a role in iron uptake, and 4) OxyR, H-NS, RcsAB, GcvA, OmpR, Fis, and LysR/PBP2 family proteins were predicted as putative regulators.

## Methods

### *K. pneumoniae* genomic sequences

The nucleotide sequences and annotations of *K. pneumoniae* Kp52.145 (NZ_FO834906, NZ_FO834904 and NZ_FO834905), HS11286 (NC_016845.1, NC_016838.1 and NC_016846.1) and NTUH-K2044 (NC_012731.1 and NC_006625.1) chromosomes and plasmids were downloaded from the NCBI RefSeq database.

### In silico identification of T6SS-encoding genes

T6SS gene clusters were identified by SecReT6 [10] and VRprofile [9] algorithms,. considering e-value ≤10^− 10^ and identity ≥70%..

Conserved domains were identified in CDD (Conserved Domain Database), PFAM (Protein Families Database), and COG (Clusters of Orthologous Groups) databases [55–57]. Sequence alignments were performed with Clustal [58] .

### Synteny analysis

Nucleotide sequences were aligned using the DoubleACT v2.0 online resourceand visualized in the Artemis Comparison Tool [59]. Syntenic regions (% identity > 87) were represented as solid red blocks.

### Computational prediction of promoter sequences and operons

Genomic regions containing 250 bp upstream each of the previously identified T6SS genes were analyzed using Bprom algorithm [60]. The Bprom algorithm identify putative binding sites for the sigma-70 factor.

### Prediction of protein binding sites in promoter sequences

The 250 bp upstream each transcription start site identified above was analyzed in the Virtual Footprint prediction tool [61]. Virtual Footprint compares query sequences to a library of Position-Weight Matrixes (PWM) from Prodoric database. Those matrixes represent known transcriptional regulators binding sites.

## Additional files

Additional file 1: Summary of Secret6 and VRprofile results contributing to the annotation of T6SS genes in KP. Table describing T6SS genes in KP genomes: old and current locus-tag, genomic coordinates, gene orientation, gene size, gene product, SecreT6 and VRprofile predictions. (XLS 56 kb)

Additional file 2: Identification and location of T6SS components in the genome of KP Kp52.145, HS11286 and NTUH-K2044. Table describing locus tag, previous annotation and indicated if T6SS components to belong in loci or are orphan. (XLS 284 kb)

Additional file 3: Multiple sequence alignment of ClpV, ClpB and ClpA proteins from *Klebsiella pneumoniae* Kp52.145**.** Green and yellow regions indicate the Clp_N domain; Red, blue and purple are the AAA, AAA2 and ClpB_D2-small domains, respectively. Black and blue boxes indicate the Walker A and Walker B domains; Arrows point to ATP binding site residues. “*”Identity of all aminoacid residues in the indicated column, “:”Similarity of pairs aligned, “.” Low similarity of pairs aligned. (PDF 58 kb)

Additional file 4: Predicted 휎70 promoter sequences of T6SS genes. Binding sites − 10, spacer region, − 35 and Transcriptional Start Site upstream the CDSs coding for the T6SS-related genes in genome of *Klebsiella pneumoniae* Kp52.145, HS11286 and NTUH-K2044. (XLS 98 kb)

Additional file 5: Putative promoter sequence of T6SS genes. 250 bp upstream those transcriptional start sites of T6SS genes in genome of *Klebsiella pneumoniae* Kp52.145, HS11286 and NTUH-K2044. (TXT 11 kb)

Additional file 6: Hypothetical binding sites for transcriptional regulators predicted in putative promoter sequence of T6SS genes in Kp52.145. Position Weight Matrix (PWM), Start and End position, Strand, Score and Sequence binding for transcriptional regulators. (XLS 55 kb)

Additional file 7: Hypothetical binding sites for transcriptional regulators predicted in putative promoter sequence of T6SS genes in HS11286. Position Weight Matrix (PWM), Start and End position, Strand, Score and Sequence binding for transcriptional regulators. (XLS 42 kb)

Additional file 8: Hypothetical binding sites for transcriptional regulators predicted in putative promoter sequence of T6SS genes in NTUH-K2044. Position Weight Matrix (PWM), Start and End position, Strand, Score and Sequence binding for transcriptional regulators. (XLS 47 kb)

## Data Availability

All data generated or analysed during this study are included in this published article and its supplementary information files.
