# Supplementary material for: Insights into Klebsiella pneumoniae type VI secretion system transcriptional regulation
Source: BMC Genomics. 2019 Jun 18;20:506. doi: 10.1186/s12864-019-5885-9 (PMC6580597; doi:10.1186/s12864-019-5885-9)
Supplement: Supplementary file 3 — Multiple sequence alignment of ClpV, ClpB and ClpA proteins from Klebsiella pneumoniae Kp52.145. Green and yellow regions indicate the Clp_N domain; Red, blue and purple are the AAA, AAA2 and ClpB_D2-small domains, respectively. Black and blue boxes indicate the Walker A and Walker B domains; Arrows point to ATP binding site residues. “*”Identity of all aminoacid residues in the indicated column, “:”Similarity of pairs aligned, “.” Low similarity of pairs aligned. (PDF 58 kb) [file 12864_2019_5885_MOESM3_ESM.pdf]

```
ClpV      MENPASLLRRLNPCCARAMEGAASLCQTRAHAEILPEHWLLKLLEQGEGDLTVLARRYEW  60
ClpB      -----MRLDRLTNKFQLALADAQSLALGHDNQFIEPLHLMSALLNQEGGSVRPLLTSAGV  55
ClpA      ---------MLNQELELSLNMAFARAREHRHEFMTVEHLLLALLSNPS--AREALEACSV  49
                    *.      ::  *  :  .   :  :   :    *  :   **.:

ClpV      DMDALWQDLLSWLDKQPRSVRH------RPQLSDHTLRLMQEAWLIASLSGEAQIRSVHL  114
ClpB      NAGKLRTDIE----QALSRLPQVEGTGGDVQPSQDLVRILNLCDKLAQKKK-DNFISSEL  110
ClpA      DLVALRQELEAFIEQTTPVLPATE-EERDTQPTLSFQRVLQRAVFHVQSSGRSEVTGANV  108
          :   *  ::      :     :            *  :     *:::  .    .. .    :. .  . .:

ClpV      LMALVEKQNLIQCDGLWPLLTLGQRQLERLRP--LLDAQSDERPPAQQEAALAQPHGGDV  172
ClpB      FVLAALESRGT----LTDLL--K------------SAGATTANV---TQAIEQMRG---  145
ClpA      LVAIFSEQESQ----AAYLL--RKHEVSRLDVVNFISHGTRKDEP---SQSS--DNS---  154
          ::     :..           **                       .   :         :    ..

ClpV      EFVGRPAGSELNADGLNPALQNALDKFTLDVTAKARDGLIDPVFGRDTEIRQMVDILSRR  232
ClpB      -------GESVN-DQGAEDQRQALKKFTVDLTERAEQGKLDPVIGRDEEIRRTIQVLQRR  197
ClpA      -------GSQPG-NEEQAGGEERMENFTTNLNQLARVGGIDPLIGREKELGRAIQVLCRR  206
                 *.   :          .: :.:** ::.   *.  *  :**::**: *:  :  :::* **

ClpV      RKNNPILVGEPGVGKTALVEGLALRIAEGNVPDALKPVSVRTLDLGLLQAGAGVKGEFEQ  292
ClpB      TKNNPVLIGEPGVGKTAIVEGLAQRIVNGEVPEGLKGRRVLALDMGALVAGAKYRGEFEE  257
ClpA      RKNNPLLVGESGVGKTAIAEGLAWRIVQGDVPEVMADCTIYSLDIGSLLAGTKYRGDFEK  266
           ****:*:** ******:.**** **.:*:**:  :    :  :**:* * **:   :*:**:

ClpV      RLKNIIEAVQQSPSPVLLFIDEAHTIIGAGNQG-GGADAANLLKPALARGELRTIAATTW  351
ClpB      RLKGVLTDLSKQEGNVILFIDELHTMVGAGKAD-GAMDAGNMLKPALARGELHCVGATTL  316
ClpA      RFKALLKQLE-QDTNSILFIDEIHTIIGAGAASGGQVDAANLIKPLLSSGKIRVIGSTTY  325
          *:*  ::   :.  .     :***** **::***    .  *   **.*::** *: *::: :.:**

ClpV      SEYKQYFERDAALERRFQMVKVDEPDDDTACLMLRGLKSRYADHHGVHITDDAVRAAVTL  411
ClpB      DEYRQYIEKDAALERRFQKVFVAEPSVEDTIAILRGLKERYELHHHVQITDPAIVAAATL  376
ClpA      QEFSNIFEKDRALARRFQKIDITEPSVEETVQIINGLKPKYEAHHDVRYTAKAVRAAVEL  385
          .*:  :  :*:* ** **** :  :  **.  :  :   ::.*** :*    ** *: *   *: **. *

ClpV      SRRYLTGRQLPDKAVDLLDTASARLRMSLDTVPEPLTRMKAQLTALAMEKQALLEDIALG  471
ClpB      SHRYIADRQLPDKAIDLIDEAASSIRMQIDSKPEELDRLDRRIIQLKLEQQALKKESDEA  436
ClpA      AVKYINDRHLPDKAIDVIDEAGARARLMPVSKRKK------------------------  420
          :  :*:  .*:*****:*::* *.:   *:    :   :

ClpV      NSARGDRLAAIEQEEIRLILALDTLETQYGQE-------------LQLTEALLACRRDIS  518
ClpB      SLKRLDMLN---EELADKERQYSVLEEEWKAEKASLSGTQTIKAELEQAKIAIEQARRVG  493
ClpA      ------------------------------------------------------------  420

ClpV      RQAE--------INDLQTALIA----VQQGNPLLGLDVDVRTVATVIADWTGVPLSSLMK  566
ClpB      DLARMSELQYGKIPELEKQLAAATQSEGKTMRLLRNKVTDAEIAEVLARWTGIPVSRMME  553
ClpA      ------------------------------------TVNVADIESVVARIARIPEKSVSR  444
                                              *     :   *:*   :  :*  . :  .

ClpV      DEQTELLSLEESLGKRVVGQEAALSAIARRLRAAKTGLTPENGPQGVFLLVGPSGTGKTE  626
ClpB      SERDKLLRMEQELHHRVIGQDEAVEAVSNAIRRSRAGLSDPNRPIGSFLFLGPTGVGKTE  613
ClpA      SDRDTLKNLGDRLKMLVFGQDKAIEALTEAIKMARAGLGHEHKPVGSFLFAGPTGVGKTE  504
          .::   *   :  :  *    *.**: *:.*::. :: :::**     :  *  *  ** :  **:*.****

ClpV      KALALADALFGGEKALITINLSEYQEPHTVSQLKGSPPGYVGYGQGGILTEAVRKRPYSV  686
ClpB      LCKTLANFMFDSDDAMVRIDMSEFMEKHSVSRLVGAPPGYVGYEEGGYLTEAVRRRPYSV  673
ClpA      VTVQLAKA---LGIELLRFDMSEYMERHTVSRLIGAPPGYVGFDQGGLLTDAVIKHPHAV  561
              **.          ::  :::**:  *  *:**:* *:*******:  :** **:**  ::*::*

ClpV      VLLDEVEKAHRDVMNLFYQVFDRGVMRDGEGREIDFRNTVILMTANLGSDLLMQLLDEQP  746
ClpB      ILLDEVEKAHPDVFNILLQVLDDGRLTDGQGRTVDFRNTVVIMTSNLGSDLIQERFGELD  733
ClpA      LLLDEIEKAHPDVFNLLLQVMDNGTLTDNNGRKADFRNVVLVMTTNAGVRETERKSIGLI  621
          :****:**** **:*:: **:* *  :  *.:**   ****.*::**:* *      .

ClpV      EASESDLHELLRPVLRGHFQPALLARFQTVI-YRPLPAGALRAIVGMKLGQVSQRLACHY  805
ClpB      ---YGHMKDLVLGVVSQNFRPEFINRIDEVVVFHPLGEKHIASIAQIQLQRLYKRLE-ER  789
ClpA      ---QQDNSPDAMDEIKKIFTPEFRNRLDNIIWFDHLSTTVIHQVVDKFIVELQVQLD-QK  677
             .       :    * *  :   *:: :: :   *     :   :.     :  .:   :*   .

ClpV      GITTTLSESLFDALTEACLLPDTGARNVDSLLNQQILPALSQQLLSHMAAGQKPRQVTLG  865
ClpB      GYEVQMSDEALKLLSANGYDPVYGARPLKRAIQQQIENPLAQQILSGELVPGKTIELVV-  848
ClpA      GVSLEVSQEARDWLATKGYDRAMGARPMGRVIQDNLKKPLANELLFGSLVDGGQVTVSL-  736
          *    :*:.   . *:         *** :       :::::    *::::*    .        : :

ClpV      YHEEEGVVMAFDEGTISDE------  884
ClpB      -KDDR-IVAVQ--------------  857
ClpA      -DSDK-NTLTYDFQSAPKHKPEAAH  759
           ..:.  . .
```
